# Socioecological Factors Influencing Sexual Health Experiences and Health Outcomes of Migrant Asian Women Living in ‘Western’ High-Income Countries: A Systematic Review

**DOI:** 10.3390/ijerph18052469

**Published:** 2021-03-03

**Authors:** Corie Gray, Gemma Crawford, Bruce Maycock, Roanna Lobo

**Affiliations:** 1 Collaboration for Evidence, Research and Impact in Public Health, School of Population Health, Curtin University, Bentley 6102, Australia; g.crawford@curtin.edu.au (G.C.); Roanna.lobo@curtin.edu.au (R.L.); 2 College of Medicine & Health, University of Exeter, Devon EX4 4PY, UK; bmaycock@iinet.net.au

**Keywords:** migrants, women, systematic review, sexual and reproductive health, socioecological model

## Abstract

Migrant health has been identified as one of the most pressing issues of the 21st century. Migration experiences are influenced by gender and gender norms and have important implications for the sexual health of migrant women. This systematic review explored socioecological factors influencing sexual health experiences and health outcomes of migrant Asian women living in “Western” high-income countries. PRISMA guidelines were followed and this study was registered with PROSPERO. Five academic databases were searched for peer-reviewed articles published between 2000 and 2019. Of 2415 studies retrieved, 17 met the selection criteria: 12 qualitative, 4 quantitative, and 1 mixed-methods study. The four levels of Bronfenbrenner’s socioecological model were applied to examine the individual, interpersonal, institutional, and societal factors of influence. Most studies (*n* = 13) reported individual level factors, focusing on knowledge and use of contraceptives. At a societal level, host country sociocultural factors, including gender and cultural norms, influenced knowledge, ability to access and utilise contraceptives, and access to health services. Findings suggest that the public health policy, practice, and research to improve the sexual health of migrant women requires greater consideration of the intersecting factors of gender, culture, and the migration process.

## 1. Introduction

Migration is a defining feature of the 21st century, acknowledged as “increasingly heterogeneous, fluid and nonlinear” (p. 2264, [1]). Previously described as “gender blind” [2], there has been a growing emphasis on gender in migration theory [3] with increased recognition that migration is influenced by gender, gender norms, and relationships. 

In 2017, Asia was the largest contributor of international migrants globally, with the majority residing in Europe, North America, and Oceania [4]. Approximately half of migrants from Asia are women [5]. Historically, migrating Asian women followed spouses; more recently, there has been an upward trend in solo migration [6]. This is in part due to wage disparities between countries; access to education and employment opportunities outside of country of birth; and increased labour demand, particularly in the health industry [6]. Consequently, women from Asia are the primary income providers for their families, shaping new gender and power ideals [3].

The term “Asian” has been used to group people by geographical origin or ancestral background. It also includes various groups of racial, ethnic, and cultural characteristics. However, such a broad term can mask considerable differences between groups and communities [7] including language, religion, cultural norms, and laws [8]. Most “Asian” cultures are considered to be patriarchal and patrilineal societies [8], though this differs by country based on cultural norms, government policy, and economic change [9]. More countries in Western Asia (e.g., the Islamic Republic of Iran, the Kingdom of Saudi Arabia, and the Republic of Yemen) and South Asia (e.g., the People’s Republic of Bangladesh, the Republic of India, and the Federal Democratic Republic of Nepal) have policies that legally discriminate against women (i.e., have a law that differentiates between men and women, for example restrictions on owning property and accessing health services) than other regions globally [10].

The “West” [11] describes perceived commonalities in countries, particularly those in the United States of America (USA), Dominion of Canada, the United Kingdom of Great Britain and Northern Ireland (UK), European countries (e.g., the French Republic, the Italian Republic, and the Kingdom of Spain), New Zealand, and the Commonwealth of Australia. Considered to have ancestry from the “West”, countries include often English-speaking, former British colonies, with Christianity being the dominant religion historically, shaping social and cultural norms and laws. Politically, “Western” countries are often regarded as liberal democracies [12], favouring individual rights and freedoms.

Differences and tensions between “Western” and “Asian” cultures can be challenging for migrants to navigate, including around gender norms and attitudes towards women’s sexuality and premarital sex [13]. Structural inequalities (for example, laws and policies that disadvantage certain population groups) faced by migrants have a significant impact on health seeking behaviour and health outcomes [14]. Given the impact that migration contexts have on health, migration has been posited as a determinant of health (Castañeda et al., 2015; Davies, Basten, and Frattini 2009).

In “Western” high-income countries (HICs), migrant women often report poorer sexual and reproductive health (SRH) outcomes and record lower utilisation of SRH services [15]. Australian research suggests women from Asia are more likely to be diagnosed with hepatitis B than Australian-born women and more likely to be diagnosed late for Human Immunodeficiency Virus (HIV) [16]. European research has found that migrant women are at increased risk of HIV and sexually transmissible infection (STIs), and access sexual health services far less than non-migrants [17].

Effectively responding to the health disparities faced by migrants remains a global pressure [18]. There is a need to understand how structural and sociocultural influences impact migrant health throughout the migration process [14]. While different frameworks exist, Bronfenbrenner’s socioecological model (SEM) is well-established in public health and has been used to challenge interventions that attempt to change individual health-related behaviour without acknowledging the broad socioecological influences that maintain or reinforce poor health [19,20]. It has utility in determining where public health interventions may be most effective for migrant sexual health outcomes [21] and supports the need for multilevel interventions that address individual (i.e., health knowledge and behaviour), interpersonal (i.e., influence of social networks), institutional (i.e., institutional policies and culture), and societal factors (i.e., public policies and social and cultural norms) [19,20,22].

This systematic review aimed to explore the sexual health experiences and sexual health outcomes of “Asian” women living in “Western” HICs using Bronfenbrenner’s (1992) SEM [23]. The review consolidates the existing literature on sexual health outcomes of “Asian” women to inform future research and public health interventions relating to their sexual health.

## 2. Methods

The Preferred Reporting Items for Systematic Reviews and Meta-Analyses (PRISMA) statement guided this review [24]. Procedures followed those used in other systematic reviews published by the research team [25,26,27,28]. The review was registered with the PROSPERO International prospective register of systematic reviews (Registration: CRD42020164726, date: 24 August 2020).

### 2.1. Study Eligibility

The review included peer reviewed, qualitative, and quantitative studies, published in English between 2000 and 2019, describing the sexual health experiences of Asian women living in “Western” HICs (inclusive of and similar to Australia). HICs were defined as those Organization for Economic Co-operation and Development (OECD) countries with Gross National Income (GNI) per capita above USD 12,236 [29]. While multiple definitions exist, in this study, “Western” countries included the USA and Canada, the UK, New Zealand, Australia, and those in Europe (e.g., France, Italy, and Spain), due to their broad historical, cultural, social, and political similarities. “Asia” was defined as the 48 countries and 3 other territories within the “Asian” continent [30]. The review included studies in the literature focusing on the sexual health of women aged over 18-years-old, born in Asian countries. Articles including younger women (under 18 years of age) in addition to women over the age of 18 were included; however, studies that included men, women from other regions, or those only under 18 years of age were excluded. “Sexual health” was used per the World Health Organization working definition, “a state of physical, mental and social well-being in relation to sexuality” [31]. The focus was common concepts and outcomes reported in public health: sex, sexuality, and sexual and reproductive rights; sexual health knowledge and attitudes; unsafe sex and STIs [32]. Only available, full-text, primary research studies were included. Search terms were selected to identify articles relating to individual and sociocultural factors that affect sexual health, guided by the levels of Bronfenbrenner’s SEM [20,23,33].

### 2.2. Search Strategy and Study Selection

Five electronic databases were searched: Medline, Scopus, ProQuest, PsychInfo, and Informit. References from grey literature (for example, reports, textbooks, and policy papers) were excluded. Reference lists of included papers were hand-searched for additional articles. Table 1 presents search terms, including keywords and MeSH index terms.

**Figure 1:**
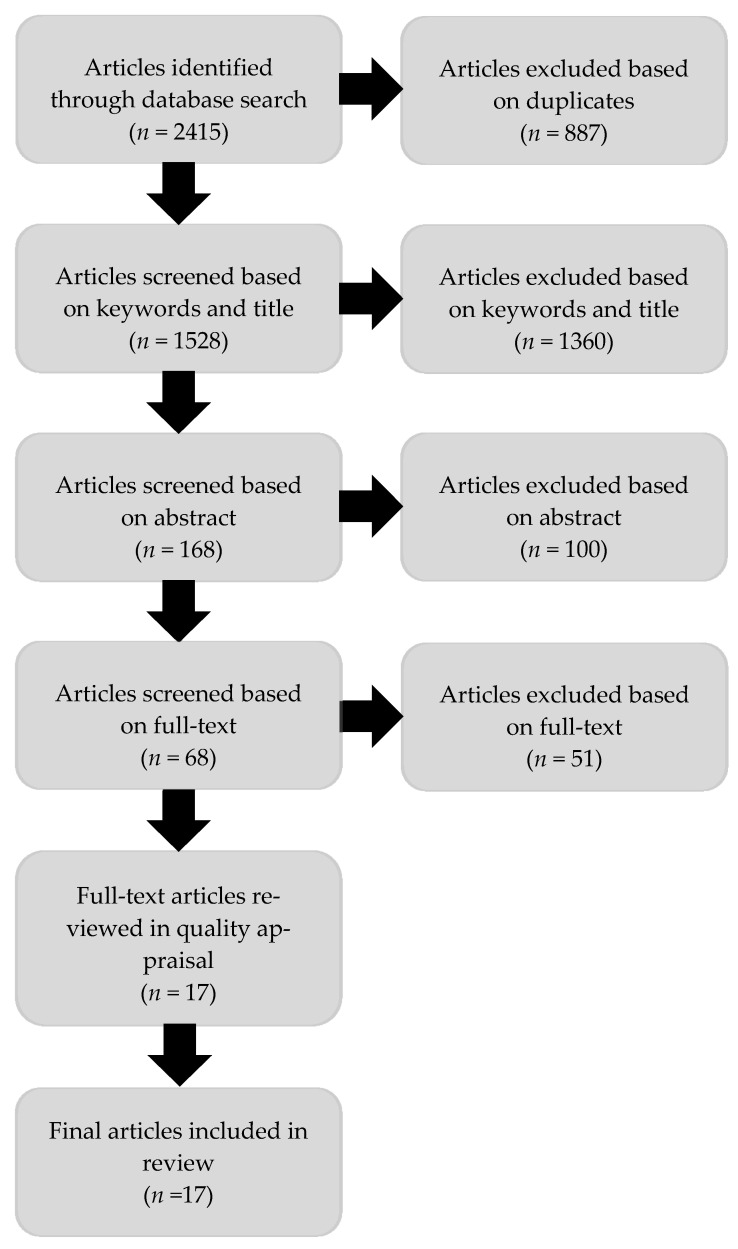
Flow diagram of review process.

Citations were downloaded into EndNote X9 citation management software [34]. Duplicate papers were removed prior to screening. Articles were initially screened by title, excluding those clearly irrelevant according to population, interest, or context [35]. Articles where the title was unclear or ambiguous were included for abstract screening. Subsequently, articles were imported into Covidence [36], a web-based software for primary screening and data extraction in the production of systematic reviews. Titles and abstracts were screened against the inclusion criteria by two reviewers. Disagreements during screening (e.g., reviewer error regarding population) were resolved through discussion. Full-text articles were reviewed by CG. Figure 1 shows the review process. Most articles excluded based on full-text were due to wrong population (i.e., not involving women, not exclusively migrants, etc.). A list of excluded full-text articles is available in Table S1.

### 2.3. Quality Appraisal and Data Extraction

Articles included in full-text screening underwent quality appraisal using the Joanna Briggs Institute (JBI) series of assessment and review instruments to assess methodological quality [37]. Quality appraisal was undertaken by CG and checked with the research team. Data were extracted by the primary researcher and entered into a modified JBI extraction table [37]. Results were reviewed and verified by the research team for consistency. Results were reported using the four levels of the SEM.

## 3. Results

Seventeen studies were included. The data extraction table is included in the Table S3. Results were categorised into the following domains:

- Individual/personal factors, such as knowledge and behaviour;
- Interpersonal relationships, including partner, family, and peer influence;
- Institutional settings and institutions, including healthcare, workplaces, and schools;
- Societal/broad social factors, including social norms and policies.

Articles at each socioecological model level are presented in Table 2, acknowledging that most articles (*n* = 9) addressed more than one SEM level.

### 3.1. Overview of Studies

Six studies were conducted in Australia [40,43,45,49,52,54], five in the USA [41,42,44,51], three in Canada [48,50,55] and Sweden [38,39,53], and one in the UK [46]. Studies included participants born in Bhutan [42], Cambodia [41], China [40,44,46,48], Iran [52,54], Malaysia [40], Japan [43], South Korea [53], and Thailand [38,39,53]. The term “Asian” was used to describe participants in five studies [45,49,50,51,55].

Twelve studies were qualitative [38,40,41,42,43,46,48,49,50,52,53,54], using interviews (*n* = 10) and focus groups (*n* = 3). Remaining studies (*n* = 4) used cross-sectional surveys [39,44,45,47]. One study used both qualitative and quantitative methods [51]. Sample size ranged from 7 to 210. Three articles presented selected data from a larger study [43,47,48].

### 3.2. Participant Characteristics

The age of participants ranged from 16 to 82 years; most studies focused on adult women <40 years. Time in host country was reported in 13 studies and ranged from <1 to 29 years. Citizenship/legal status in host country was reported in four studies with categories: citizen, permanent resident, refugee and asylum seeker, spousal, student, tourist and work visas, and illegal stays. Ten studies reported marital status: single or married. No study reported sexuality.

### 3.3. SEM Level—Individual

Thirteen articles described sexual health knowledge, contraception use, and attitudes towards disease prevention. Studies reported low STI and contraceptive knowledge, but positive attitudes towards disease prevention and contraceptive use.

#### 3.3.1. Sexual Education and Knowledge

Nine studies described women’s access to sexual education and knowledge of STI and HIV transmission modes and prevention. Consistently, little education on sexual health in the home country was reported along with low levels of HIV and/or other STI knowledge and awareness [40,46,49,50]. Four studies measured HIV and/or other STI knowledge, reporting both high [39,45] and low levels of knowledge and awareness [44,47]. Knowledge of HIV transmission was high among Thai and Chinese sex workers in Australia [45]. Almost all women correctly identified that HIV was transmitted by anal/vaginal unprotected sexual intercourse (93–99%); from mother-to-child (93–88%); and via needles (94–92%). Participants were less certain about other risks. In a survey by Akerman and colleagues, Thai women living in Sweden reported high levels of knowledge on STI and pregnancy prevention; however, knowledge was not tested [39].

Conversely, a survey with Asian women in Canada found low knowledge and awareness of HIV and other STIs. Almost a third (31%) of women had never heard of HIV and half had never heard of STIs [47]. In a survey with Chinese women in the USA, Nguyen and colleagues found less than one-fifth had heard of HPV (19%) or of a vaccine to prevent cervical cancer (19%).

Three qualitative studies also suggested low STI awareness. In interviews with Asian women living with HIV in Canada, Hawa et al. (2017) found that most reported little knowledge of HIV prior to their diagnosis. Likewise, interviews with young Muslim women in Australia by Wray et al. (2014) suggested low awareness of STIs. Burke et al. (2015) conducted interviews with Cambodian mothers in the USA, finding participants were uncertain about what diseases the HPV vaccination protected their daughters against. Beliefs included that the vaccine protected against ovarian cancer or that it protected from all STIs; women were also uncertain regarding the duration of protection [41].

#### 3.3.2. Contraception Use

Seven studies described the use of contraceptives. In qualitative studies by Dhar et al. (2017) and Inoue et al. (2016), women described the importance of contraceptives to prevent unwanted pregnancies. Japanese women living in Australia discussed the use of dual or triple protection—male condoms, withdrawal method, and the contraceptive pill; or the use of abstinence to protect against pregnancy [43]. By contrast, Micollier et al. (2017) found Chinese women in Canada considered both pregnancy and STI prevention to be of equal importance.

Five qualitative studies reported women had limited awareness of contraception options, and were most commonly aware of the contraceptive pill [40], the male condom [42,46], or both [43,49]. By contrast, Chinese women living in the UK described mandatory use of the intrauterine device (IUD) after birth and little knowledge of other contraceptives [46]. Asian women living with HIV described the stigma attached to purchasing or using male condoms, such as perceptions of multiple sexual partners or of being a sex worker [50].

One article measured contraception use. Comparing two surveys conducted with Thai and Chinese sex workers in 1993 and 2003 in Australia, Pell et al. (2006) found that condom use had increased for vaginal (52% vs. 85%), oral (40% vs. 66%), and anal sex (20% vs. 78%).

#### 3.3.3. Attitudes towards Prevention

Four out of five studies reported predominantly positive attitudes towards prevention. Gagnon et al. (2010) found that a majority (80%) of Asian women believed it was acceptable for AIDS to be discussed in school, and 60% supported teaching teenagers to use condoms to avoid HIV. Hawa et al. (2017) and Akerman et al. (2017) also reported positive attitudes towards sexual education. Burke et al. (2015) found that Cambodian mothers expressed positive attitudes towards HPV prevention, with a desire to protect their daughters from future health issues.

Nguyen et al. (2012) reported that Chinese women had negative attitudes towards the HPV vaccine. One-third (31%) of women wanted the HPV vaccine for their daughter/granddaughter at little/no cost; this proportion decreased to 23% when there was a cost involved. One-fifth (20%) of participants believed the HPV vaccine would lead to their daughter/granddaughter becoming more sexually active [44].

### 3.4. SEM Level—Interpersonal

Four studies described women’s relationships with their sexual partner, with women reporting a belief they were unequal in relationships. Condom use, sexual intercourse, and reproductive outcomes were reported as often regulated by men. Gagnon et al. (2010) reported Asian men were more likely to report higher sexual decision-making power. In this study, women who had high sexual decision-making power were significantly more likely to have heard of STIs (25.5%, CI 3.0–48), and felt they could ask their partner to use a condom (28.2% CI 4.7–51.8), compared to women with low sexual decision-making power. Hawa et al. (2017) and Micollier et al. (2017) described women’s difficulties negotiating spousal condom use. Findings from both studies suggested that women were expected to listen to their husbands, who were described in one study as being “central in her world” (p. 952, [50]).

Using mixed methods, Raj et al. (2005) explored experiences of IPV among Asian women in the USA. Women experiencing IPV were significantly more likely to report negative health outcomes such as an unwanted pregnancy (95% CI 1.33–8.66). Three-quarters of interview participants reported sexual IPV while 40% had reported unwanted pregnancy due to sexual assault. Women also reported being coerced to terminate a pregnancy [51].

### 3.5. SEM Level—Institutions

Eight articles described women’s access to health services. Studies suggested lack of clarity navigating sexual health services, and language barriers in accessing and understanding services.

#### 3.5.1. Navigating the Health System

Akerman et al. (2017) and Dhar et al. (2017) found women were uncertain about accessing health services in the host country. Akerman and colleagues conducted interviews with Thai women living in Sweden, who described limited experience accessing the Swedish healthcare system, despite stated needs. Women reported uncertainty about services. As such, women often purchased contraceptives when travelling back to Thailand, or asked Thai friends and family to purchase contraceptives. Male partners played a critical role in enabling women to access health services, providing information on SRH and booking appointments [38]. Dhar et al. (2017) found young Muslim women born in Bhutan held misconceptions around accessing health services in the USA. Women held assumptions that unmarried women were not allowed to access SRH services; that a parent was required if under the age of 18 years; or that if married, male partners had to provide consent for a woman to access SRH services unaccompanied [42].

#### 3.5.2. Language

Seven articles described language challenges. Japanese women reported difficulty discussing hormonal contraception with their general practitioner (GP), consequently using other methods such as condoms or withdrawal [43]. Likewise, Iranian women participating in focus groups reported challenges in discussing sexual health with GPs, due to differences in language used to describe genitals and sexual behaviour [54]. Cultural misunderstandings were also discussed by Chinese women in relation to contraceptives and how they were described [46]. Lack of certainty regarding contraceptives or concerns about their long-term effects resulted in removal of intrauterine contraceptive devices or non-adherence [46]. Cambodian mothers described confusion around HPV vaccination, reporting difficulties understanding English material [41]. Akerman et al. (2017) reported Swedish male sexual partners were often used as interpreters for Thai women despite not being able to speak Thai.

Two quantitative studies demonstrated the impact of English proficiency on knowledge and attitudes related to healthcare access. Using a cross-sectional survey with Chinese women, Nguyen et al. (2012) found ability to speak English was significantly associated with supporting daughters/granddaughters to receive the HPV vaccination (OR = 10.7, 95% CI (1.8, 62.3), *p* = 0.008) and believing HPV vaccine could prevent an STI (OR = 7.6, 95% CI (1.1, 55. 3), *p* = 0.045). Similarly, preference to respond to a survey in English was significantly associated with some domains of HIV and STI knowledge, compared to those who did not answer in English [47].

### 3.6. SEM Level—Societal

Five articles reported on societal attitudes towards women, finding an emphasis on virginity and requiring women to control their sexuality.

#### Attitudes towards Women and Women’s Sexuality

Five articles explored attitudes towards women and women’s sexuality. Three articles described the value of women’s virginity within their community including the consequences of sex before marriage. Focus groups with international students from China and Malaysia reported the importance of female virginity; consequently, women were discouraged from engaging in sex before marriage [40]. Unmarried Bhutanese women reported discomfort seeking sexual health information for fear of judgment of premarital sex [42]. Consequences of sex before marriage included being disowned by the family, or increased coercion to marry [42]. In a study by Wray and colleagues, young Muslim women described how their community regulated sexual experience and knowledge. Women who engaged in premarital sex were described as “fallen women”. Physical violence and coercion into marriage were considered acceptable punishment within the community [49].

In studies by Lindblad et al. (2008), Khoei et al. (2008), and Wray et al. (2014), women described expectations that they should be sexually submissive. In interviews with adoptees from South Korea or Thailand living in Sweden, participants described perceptions that Asian women were expected to be “sexually obedient” and that they were more likely to be grateful for sexual invitations. Women described attitudes towards Asian women resulting in sexual harassment, including unwanted touch [53]. Similarly, Wray et al. (2014) found expectations of “sexual obedience” by young Muslim women who reported having to provide spouses with sex “whenever he wanted”. In interviews and focus groups with Iranian women, participants described sexual submission as a religious duty, and part of being an idealised Muslim wife [52].

### 3.7. Recommendations for Research, Practice, and Policy

Eleven studies made recommendations, all relating to practice—none related to research or policy. For example, four studies made recommendations for clinical practice, namely free health screenings [38,39,45,51], and three suggested the provision of health information or health services in other languages [43,44].

## 4. Discussion

This systematic review included 17 studies, published between 2000 and 2019, which reported on the sexual health experiences and outcomes of Asian women living in “Western” HICs. Findings were mapped to SEM constructs [20,23] to identify individual, interpersonal, institutional, and societal factors affecting migrant women’s sexual health

While some studies could be mapped to more than one SEM level, the majority of studies (*n* = 13) focused on individual level factors, primarily women’s sexual health knowledge. Consistent with the broader literature, these studies demonstrated low awareness of STIs across countries of birth [56,57]. Women’s experiences of sexual and reproductive health were shaped by patriarchy and heteronormativity. For example, sexual health knowledge, and ability to access and use contraceptives, were regulated by males. In many cases, women were expected to adjust personal sexual desires in accordance with cultural and religious ideologies [49]. Similar experiences have been reported with broader groups of migrant women [56,57] with implications for women’s ability to access sexual health education and contraceptives.

At an interpersonal and societal level, there was some evidence that women experienced difficulty negotiating condom use, with expectations that women were to be sexually obedient and submissive. Women also described a range of negative consequences in instances of unwanted pregnancies or sex before marriage including physical and sexual violence and reproductive coercion. Women frequently reported that they lacked sexual agency and power [47], with gender dynamics within relationships acting as deterrents to sexual health knowledge and contraception use [48]. Whilst many studies recommended sexual health education for migrant women, the SEM suggests interventions focusing on individual health behaviour (such as education) may not be effective on their own given considerable influences on knowledge, attitudes, and behaviour experienced at the interpersonal and societal level. Previous research has demonstrated an absence of reported societal interventions to address STIs amongst migrants [26]. Women’s health, including their ability to develop sexual health literacy and navigate condom use, is strongly affected by sociocultural factors including social justice, gender equality, and human rights [58,59]. Intersectionality theory presents a way to understand the way in which an individual’s social and political characteristics interact [60]. It views a person as holistic and examines the multiple axes on which these factors interconnect and are interactive [61]. The intersectionality framework has been recommended as an approach for migrant women’s sexual health [56,61,62], as it considers factors of culture, race, migration status, sexuality, and gender that contribute to health inequalities [63]. Future studies and interventions could assess the ways in which societal factors influence migrant women’s sexual health [14], to better inform public health interventions beyond an individual focus, using the SEM to guide reporting.

Women experienced institutional barriers accessing sexual health services and education, consistent with those reported in previous reviews [15,25]. To overcome such barriers, studies have recommended health services ensure diversity of staff (including culture and gender) and cultural competency training for staff [15,64,65]. There are also calls for culturally responsive health care systems [15] that can adapt to population mobility and transnationalism [66]. In Australia, this is consistent with other reviews [15,67]. This may require the adoption of a national culturally responsiveness framework developed by government and civil society, which should include explicit considerations of resourcing, targets, and indicators [68]. Adequate funding and support for migrant community healthcare workers, including meaningful involvement in decision-making, has shown to be effective in improving migrant health outcomes [69].

Few studies explored women’s experience of migration, despite recognition of the migration process as a determinant of health [14,70]. Zimmerman and colleagues describe the migration process as a complex, multistage cycle that can be entered numerous times, and argue that public health interventions must target each stage of the cycle: pre-travel, travel, and arrival in the destination country [71]. There are opportunities for social research to examine how migration impacts experiences of sexual health and sexual health outcomes along the migration process, informed by gender and migration theories. We encourage public health policymakers and advocates to pay greater attention to the effects of transnationalism (how migrants maintain ties to multiple countries) on access to health services and sexual health outcomes [66], and incorporate transnational perspectives in policy and intervention development. There is also a need for greater consideration of how non-health policies influence migrant health outcomes, requiring meaningful collaboration between migration and health systems [72]. The Health in All Policies approach, as adopted in South Australia, provides one way of developing intersectoral action to improve health outcomes [73].

The findings of this review highlight significant intersections between gender, culture, and health, supporting calls for more tailored research and interventions [74]. We recommend the use of conceptual and methodological frameworks such as transnationalism, intersectionality, SEM, and social determinants of health to better explore the intersection between gender, migration, and sexual and reproductive health inequalities. As with previous research [26], effective sexual health interventions for migrant populations are limited in HICs, an important consideration for those working within health promotion, education, and service delivery. Participatory approaches that build on community strengths are recommended [75,76].

### 4.1. Study Design and Reporting Limitations of Included Studies

Most studies reported methodological limitations including self-selection bias, self-reporting measures and social desirability, convenience sampling, small sample sizes, and lack of generalisability. Two studies did not report ethical approval. While most studies were conducted in a language from the participants’ country of birth, some did not report on survey translation or translation of qualitative data. In studies using an interpreter, their role was unclear. Two studies did not report on language used. Future quantitative studies should consider random sampling where possible, in addition to larger sample sizes. Surveys should be tested for validity, reliability, and cultural acceptability [77] to ensure generalisability. Qualitative studies should clearly report on the role of the researchers and interpreters during interviews, particularly how their use may influence willingness to disclose sensitive information. The COREQ provides one way of reporting qualitative research [78].

Many studies focused on specific countries of birth, which provided a more in-depth exploration of issues. However, not all Asian countries were represented; future studies could examine countries of birth not reported in this study. Additionally, larger population studies across all countries of birth may reveal similarities and differences, assisting to identify areas for intervention, research, and policy change. While migration was a focus, few studies reported on migration demographics which may influence women’s access to sexual health services and sexual health outcomes in the host country. One recommendation for policymakers is to work to harmonise national and global surveillance data using consistent measures and identifiers including country of birth, year of arrival, and visa status as starting points [79]. Peak agencies for those working with people from migrant backgrounds should be consulted for their input to ensure items are appropriate and will yield culturally sensitive and beneficial data.

### 4.2. Strengths and Limitations

This systematic review is the first to report on the sexual health experiences and outcomes of Asian women living in “Western” HICs. The review captured 19 years of peer-reviewed literature across five databases, providing a broad scope. To minimise error, two reviewers screened abstracts and full texts, and a team approach was adopted to finalise article selection. The application of the SEM facilitated understanding of how sociocultural factors impacted on individual outcomes and assisted to identify research gaps.

Included studies were peer-reviewed and in English. Grey literature was not included. We acknowledge the grey literature and non-English articles may yield valuable information on migrant women’s experiences. Meta-analysis was not conducted due to the small number of quantitative studies identified and the heterogeneity between studies. Given the limited studies on any one country of birth, we were unable to make comparisons between countries of birth or country-specific recommendations. The review was restricted to women born in Asian countries; however, we recognise the large degree of heterogeneity between and within these countries, including language, religion, and cultural norms. It is acknowledged that “Asian” is a broad term; policy and service delivery would be better served if future studies made more nuanced consideration of specific groups, countries, and regions. We recognise the socially constructed nature of the nomenclature around “Asia” and the “West” and how this may conceal differences between countries and cultures. Despite limitations, this review provides a contemporary snapshot of sexual health issues for Asian women living in “Western” HICs.

## 5. Conclusions

Asian migrant women in “Western” HICs experience poorer sexual health outcomes, lower utilisation of health services, and low levels of sexual health knowledge in country of arrival. Findings from this review highlighted a predominance of individual factors reported in the literature that influence sexual health outcomes, such as knowledge and attitudes. The review revealed limited consideration of broader interpersonal, institutional, and societal factors that influence health. Findings also suggest little attention to the nuanced experience of migration, or how migration impacts experiences of sexual health and sexual health outcomes across different phases of the migration journey. Critically, we suggest that to improve sexual health outcomes for migrant women, public health research and practice requires concerted efforts to address “upstream” societal and institutional factors that influence individual knowledge, attitudes, and behaviour, such as health service access and gender equity.

## Figures and Tables

**Table 1:** Example of search terms used in Medline.

**Concept 1 (Mobility)**
“Transient and Migrants”
“Emigrants and Immigrants”
Refugees
“International student”
Human migration
**Concept 2 (Sex)**
“Sexual health”
Sex education
HIV infections
Sexual Behaviour
Sexual Transmitted Diseases
Unsafe sex
**Concept 3 (Gender)**
Gender
Woman
Women
Feminist
Feminine
Female
**Concept 4 (Asia)**
All 48 countries, 3 territories included *
Asia, central
Asia, western
Asia
Asia, southeastern
Asia, northern

* A full list of countries and territories is provided in Supplementary Material 2.

**Table 2:** Overview of articles by socioecological model (SEM) level [23].

SEM Level	Themes Included	Number of Articles * *(n)*	Citations
Individual	Sexual health knowledge; contraception and condom use; attitudes towards prevention	13	[38,39,40,41,42,43,44,45,46,47,48,49,50]
Interpersonal	Sexual relationships and relationship power	4	[47,48,50,51]
Institutions	Health service access	8	[38,41,42,43,44,46,47,52]
Societal	Attitudes towards women	5	[40,42,49,52,53,54].

* Note: Seven studies addressed two levels [38,41,43,44,46,48,50]. Two studies addressed three levels [42,47].

## Data Availability

Not applicable.

## Supplementary Materials

The following are available online at https://www.mdpi.com/1660-4601/18/5/2469/s1, Table S1: Full list of excluded articles; Supplementary Material 2: List of Asian countries included; Table S3: Data extraction summary.
