# Supplementary material for: Socioecological Factors Influencing Sexual Health Experiences and Health Outcomes of Migrant Asian Women Living in ‘Western’ High-Income Countries: A Systematic Review
Source: Int J Environ Res Public Health. 2021 Mar 3;18(5):2469. doi: 10.3390/ijerph18052469 (PMC7967613; doi:10.3390/ijerph18052469)
Supplement: Supplementary file 1 [file ijerph-18-02469-s001.pdf]

Table S1. Articles excluded during full-text screening.

Study	Reason for exclusion
Agu et al. (2016) [1]	Wrong population group
Ahmed et al. (2017) [2]	Wrong population group
Ahmadi (2003) [3]	Not a primary study
Akerman et al. (2019) [4]	Wrong population group
Alessi et al. (2017) [5]	Wrong population group
Ati (2012) [6]	Not a peer-reviewed article
Barkho et al. (2011) [7]	Sexual health outcomes not reported
Bjerke et al. (2010) [8]	Wrong population group
Black et al. (2013) [9]	Sexual health outcomes not reported
Brotto et al. (2008) [10]	Wrong population group
Bungay et al. (2012) [11]	Wrong population group
Chen et al. (2015) [12]	Wrong population group
Dua (2007) [13]	Not a primary study
Espiritu (2003) [14]	Not a peer-reviewed article
Fisher et al. (2003) [15]	Wrong population group
Gao et al. (2016) [16]	Wrong population group
Goldenberg et al. (2015) [17]	Wrong population group
Gray et al. (2019) [18]	Wrong population group
Hawkey et al. (2018) [19]	Wrong population group
Hawkey et al. (2017) [20]	Wrong population group
Homma et al. (2013) [21]	Wrong population group
Huang (2017) [22]	Wrong population group
Huang et al. (2008) [23]	Wrong population group
Kim (2005)	Not a peer-reviewed article
Kott (2011) [24]	Wrong population group
Kteily-Hawa (2014) [25]	Not a peer-reviewed article
Lee et al. (2012) [26]	Wrong population group
Lee et al. (2008) [27]	Wrong population group
Lin et al. (2005) [28]	Wrong population group
Loeber (2008) [29]	Wrong population group
Lu et al. (2017) [30]	Wrong country of residence
Manderson et al. (2002) [31]	Wrong population group
Maticka-Tyndale et al. (2007) [32]	Wrong population group
Metusela et al. (2017) [33]	Wrong population group
Nemoto et al. (2007) [34]	Wrong population group
Newnham et al. (2011) [35]	Wrong population group
Porter et al. (2012) [36]	Wrong country of residence
Roy (2012) [37]	Not a primary study
Swaleshwarker et al. (2013) [38]	Wrong population group
Shimaoka (2010) [39]	Not a peer-reviewed article
Shirpak et al. (2007) [40]	Wrong population group
Simon-Kumar (2009) [41]	Not a primary study
Sinding et al. (2017) [42]	Wrong population group
Sou et al. (2015) [43]	Wrong population group
Tung et al. (2013) [44]	Wrong population group
Ussher et al. (2017) [45]	Wrong population group
Venkataramani-Kothari (2007) [46]	Not a peer-reviewed article
Vlassoff et al. (2011) [47]	Wrong population group

Woez-Stirling et al. (2001) [48]	Wrong population group
Zhou (2012) [49]	Wrong population group
Zhou (2017) [50]	Wrong population group
Zhou et al. (2016) [51]	Wrong population group

1. Agu, J.; Lobo, R.; Crawford, G.; Chigwada, B. Migrant sexual health help-seeking and experiences of stigmatization and discrimination in Perth, Western Australia: Exploring barriers and enablers. *International Journal of Environmental Research and Public Health* **2016**, *13*, doi:10.3390/ijerph13050485.
2. Ahmad, F.; Smylie, J.; Omand, M.; Cyriac, A.; O'Campo, P. South Asian immigrant men and women and conceptions of partner violence. *Journal of Immigrant and Minority Health* **2017**, *19*, 57-66.
3. Ahmadi, N. Rocking sexualities: Iranian migrants' views on sexuality. *Archives of Sexual Behavior* **2003**, *32*, 317-326.
4. Akerman, E.; Larsson, E.C.; Essen, B.; Westerling, R. A missed opportunity? Lack of knowledge about sexual and reproductive health services among immigrant women in Sweden. *Sexual & reproductive healthcare : official journal of the Swedish Association of Midwives* **2019**, *19*, 64-70.
5. Alessi, E.J.; Kahn, S.; Van Der Horn, R. A Qualitative Exploration of the Premigration Victimization Experiences of Sexual and Gender Minority Refugees and Asylees in the United States and Canada. *Journal of Sex Research* **2017**, *54*, 936-948, doi:10.1080/00224499.2016.1229738.
6. Ati, R. Intra family sexual abuse (IFSA) in the South Asian Indian family system: "A comprehensive survey of the literature and critical analysis". *Dissertation Abstracts International: Section B: The Sciences and Engineering* **2012**, *73*, 3255.
7. Barkho, E.; Fakhouri, M.; Arnetz, J.E. Intimate partner violence among Iraqi immigrant women in Metro Detroit: A pilot study. *Journal of Immigrant and Minority Health* **2011**, *13*, 725-731, doi:10.1007/s10903-010-9399-4.
8. Bjerke, S.E.Y.; Holter, E.; Vangen, S.; Stray-Pedersen, B. Sexually transmitted infections among Pakistani pregnant women and their husbands in Norway. In *International Journal of Women's Health*, 2010; Vol. 2, pp 303-309.
9. Black, B.M.; Chiodo, L.M.; Weisz, A.N.; Elias-Lambert, N.; Kernsmith, P.D.; Yoon, J.S.; Lewandowski, L.A. Iraqi American refugee youths' exposure to violence: relationship to attitudes and peers' perpetration of dating violence. *Violence Against Women* **2013**, *19*, 202-221.
10. Brotto, L.A.; Chou, A.Y.; Singh, T.; Woo, J.S.T. Reproductive Health Practices Among Indian, Indo-Canadian, Canadian East Asian, and Euro-CanadianWomen: The Role of Acculturation. *Journal of Obstetrics and Gynaecology Canada* **2008**, *30*, 229-238, doi:10.1016/S1701-2163(16)32759-1.
11. Bungay, V.; Halpin, M.; Halpin, P.F.; Johnston, C.; Patrick, D.M. Violence in the massage parlor industry: experiences of Canadian-born and immigrant women. *Health Care for Women International* **2012**, *33*, 262-284.
12. Chen, W.T.; Guthrie, B.; Shiu, C.S.; Wang, L.; Weng, Z.; Li, C.S.; Lee, T.S.H.; Kamitani, E.; Fukuda, Y.; Luu, B.V. Revising the American dream: How Asian immigrants adjust after an HIV diagnosis. *Journal of Advanced Nursing* **2015**, *71*, 1914-1925, doi:10.1111/jan.12645.
13. Dua, E. Exclusion through inclusion: Female Asian migration in the making of Canada as a white settler nation. *Gender, Place and Culture* **2007**, *14*, 445-466, doi:10.1080/09663690701439751.
14. Espiritu, Y.L. *Home bound: Filipino American lives across cultures, communities, and countries*; 2003.
15. Fisher, J.A.; Bowman, M.; Thomas, T. Issues for South Asian Indian patients surrounding sexuality, fertility, and childbirth in the US health care system. *Journal of the American Board of Family Practice* **2003**, *16*, 151-155.
16. Gao, H.; Okoror, T.A.; Hyner, G.C. Focus Group Study of Chinese International Students' Knowledge and Beliefs About HPV Vaccination, Before and After Reading an Informational Pamphlet About Gardasil®. *Journal of Immigrant and Minority Health* **2016**, *18*, 1085-1092, doi:10.1007/s10903-016-0349-7.
17. Goldenberg, S.M.; Liu, V.; Nguyen, P.; Chettiar, J.; Shannon, K. International migration from non-endemic settings as a protective factor for HIV/STI risk among female sex workers in Vancouver, Canada. *Journal of Immigrant & Minority Health* **2015**, *17*, 21-28.
18. Gray, C.; Lobo, R.; Narciso, L.; Oudih, E.; Gunaratnam, P.; Thorpe, R.; Crawford, G. Why I Can't, Won't or Don't Test for HIV: Insights from Australian Migrants Born in Sub-Saharan Africa, Southeast Asia and Northeast Asia. *International Journal of Environmental Research & Public Health [Electronic Resource]* **2019**, *16*, 21.
19. Hawkey, A.J.; Ussher, J.M.; Perz, J. Regulation and Resistance: Negotiation of Premarital Sexuality in the Context of Migrant and Refugee Women. *Journal of Sex Research* **2018**, *55*, 1116-1133.

20. Hawkey, A.J.; Ussher, J.M.; Perz, J.; Metusela, C. Experiences and constructions of menarche and menstruation among migrant and refugee women. *Qualitative Health Research* **2017**, *27*, 1473-1490.
21. Homma, Y.; Saewyc, E.M.; Wong, S.T.; Zumbo, B.D. Sexual health and risk behaviour among East Asian adolescents in British Columbia. *Canadian Journal of Human Sexuality* **2013**, *22*, 13-24.
22. Huang, Y. Sexuality and everydayness in a transnational context: toward a re-imagined West-China relationship? *Culture, Health and Sexuality* **2017**, *19*, 667-679, doi:10.1080/13691058.2017.1298842.
23. Huang, Z.J.; Wong, F.Y.; De Leon, J.M.; Park, R.J. Self-reported HIV testing behaviors among a sample of southeast Asians in an urban setting in the United States. *AIDS Education & Prevention* **2008**, *20*, 65-77.
24. Kott, A. South Asians living in Britain diverge over views on first sex. *Perspectives on Sexual and Reproductive Health* **2011**, *43*, 208-209.
25. Kteily-Hawa, R. HIV vulnerability amongst South Asian immigrant women in Toronto. *Dissertation Abstracts International Section A: Humanities and Social Sciences* **2014**, *75*, No Pagination Specified.
26. Lee, J.; Hahm, H.C. HIV risk, substance use, and suicidal behaviors among Asian American lesbian and bisexual women. *AIDS Education and Prevention* **2012**, *24*, 549-563, doi:10.1521/aeap.2012.24.6.549.
27. Lee, S.J.; Brooks, R.A.; Newman, P.A.; Seiden, D.; Sangthong, R.; Duan, N. HIV vaccine acceptability among immigrant Thai residents in Los Angeles: a mixed-method approach. *AIDS Care* **2008**, *20*, 1161-1168.
28. Lin, P.; Simoni, J.M.; Zemon, V. The health belief model, sexual behaviors, and HIV risk among Taiwanese immigrants. *AIDS Education and Prevention* **2005**, *17*, 469-483, doi:10.1521/aeap.2005.17.5.469.
29. Loeber, O. Sexual and reproductive health issues of Turkish immigrants in the Netherlands. *European Journal of Contraception and Reproductive Health Care* **2008**, *13*, 330-338, doi:10.1080/13625180802303626.
30. Lu, C.; McGinn, M.K.; Xu, X.; Sylvestre, J. Living in Two Cultures: Chinese Canadians' Perspectives on Health. *Journal of Immigrant & Minority Health* **2017**, *19*, 423-429.
31. Manderson, L.; Kelaher, M.; Woelz-Stirling, N.; Kaplan, J.; Greene, K. Sex, contraception and contradiction among young Filipinas in Australia. *Culture, Health & Sexuality* **2002**, *4*, 381-391.
32. Maticka-Tyndale, E.; Khosro Refaie, S.; Chinichian, M. Providing for the Sexual Health Needs of Canadian Immigrants: The Experience of Immigrants from Iran. *Canadian Journal of Public Health* **2007**, *98*, 183-186.
33. Metusela, C.; Ussher, J.; Perz, J.; Hawkey, A.; Morrow, M.; Narchal, R.; Estoesta, J.; Monteiro, M. "In My Culture, We Don't Know Anything About That": Sexual and Reproductive Health of Migrant and Refugee Women. *International Journal of Behavioral Medicine* **2017**, *24*, 836-845, doi:http://dx.doi.org/10.1007/s12529-017-9662-3.
34. Nemoto, T.; Iwamoto, M.; Morris, A.; Yokota, F.; Wada, K. Substance use and sexual behaviors among Japanese tourists, students, and temporary workers in Honolulu, Hawaii. *AIDS Education & Prevention* **2007**, *19*, 68-81.
35. Newnham, J.P.; Sahota, D.S.; Zhang, C.Y.; Xu, B.; Zheng, M.; Doherty, D.A.; Li, S.; Chung, T.; Hu, Y. Preterm birth rates in Chinese women in China, Hong Kong and Australia - The price of Westernisation. *Australian and New Zealand Journal of Obstetrics and Gynaecology* **2011**, *51*, 426-431, doi:10.1111/j.1479-828X.2011.01327.x.
36. Porter, M.; Poerwandari, K. Living culture and making relationships: Mothers and daughters negotiate sexuality in Indonesia and Canada. In *Advances in Gender Research*, 2012; Vol. 16, pp 61-89.
37. Roy, D. South Asian Battered Women's Use of Force Against Intimate Male Partners: A Practice Note. *Violence Against Women* **2012**, *18*, 1108-1118, doi:10.1177/1077801212461431.
38. Sawleshwarkar, S.; Kakar, S.R.; Jones, R.; Lagios, K.; Mindel, A.; Hillman, R.J. Indian-born patients attending a sexual health clinic in Australia have differing characteristics to their Australian-born counterparts. *Internal Medicine Journal* **2013**, *43*, 1327-1330.
39. Shimaoka, J. The effect of acculturation on openness of sexual communication: A survey of Japanese international students residing in the United States. *Dissertation Abstracts International: Section B: The Sciences and Engineering* **2010**, *71*, 2745.
40. Shirpak, K.R.; Maticka-Tyndale, E.; Chinichian, M. Iranian Immigrants' perceptions of sexuality in Canada: A symbolic interactionist approach. *The Canadian Journal of Human Sexuality* **2007**, *16*, 113-128.
41. Simon-Kumar, R. The 'problem' of Asian women's sexuality: Public discourses in Aotearoa/New Zealand. *Culture, Health & Sexuality* **2009**, *11*, 1-16.
42. Sinding, C.; Zhou, Y.R. Intimacy, identity and relationship in the accounts of Chinese immigrants to Canada: the contribution of narrative analysis. *Culture, Health and Sexuality* **2017**, *19*, 653-666, doi:10.1080/13691058.2016.1271142.

43. Sou, J.; Shannon, K.; Li, J.; Nguyen, P.; Strathdee, S.A.; Shoveller, J.; Goldenberg, S.M. Structural determinants of inconsistent condom use with clients among migrant sex workers: Findings of longitudinal research in an urban canadian setting. *Sexually Transmitted Diseases* **2015**, *42*, 312-316, doi:10.1097/OLQ.0000000000000276.
44. Tung, W.C.; Lu, M.; Cook, D.M. HIV/AIDS knowledge and attitudes among chinese college students in the US. *Journal of Immigrant and Minority Health* **2013**, *15*, 788-795, doi:10.1007/s10903-012-9716-1.
45. Ussher, J.M.; Perz, J.; Metusela, C.; Hawkey, A.J.; Morrow, M.; Narchal, R.; Estoesta, J. Negotiating discourses of shame, secrecy, and silence: Migrant and refugee women's experiences of sexual embodiment. *Archives of Sexual Behavior* **2017**, *46*, 1901-1921.
46. Venkataramani-Kothari, A. Understanding South Asian immigrant women's experiences of violence. In *Body Evidence: Intimate violence against south asian women in America*, 2007; pp. 11-23.
47. Vlassoff, C.; Ali, F. HIV-related stigma among South Asians in Toronto. *Ethnicity and Health* **2011**, *16*, 25-42, doi:10.1080/13557858.2010.523456.
48. Woez-Stirling, N.M., L Kelaher, M Benedicto, A. Young women in conflict: Filipinas growing up in Australia. Oxford: Carfax: 2001.
49. Zhou, Y.R. Changing behaviours and continuing silence: Sex in the post-immigration lives of mainland Chinese immigrants in Canada. *Culture, Health and Sexuality* **2012**, *14*, 87-100, doi:10.1080/13691058.2011.626454.
50. Zhou, Y.R. ‘Race’ and HIV vulnerability in a transnational context: the case of Chinese immigrants to Canada. *Culture, Health and Sexuality* **2017**, *19*, 695-708, doi:10.1080/13691058.2016.1192221.
51. Zhou, Y.R.; Majumdar, B.; Vattikonda, N. Culture, but more than culture: an exploratory study of the HIV vulnerability of Indian immigrants in Canada. *Culture, Health and Sexuality* **2016**, *18*, 1067-1080, doi:10.1080/13691058.2016.1162328.

Supplementary material 2 – Asian countries included in search

1. China
2. India
3. Kazakhstan
4. Saudi Arabia
5. Iran
6. Mongolia
7. Indonesian
8. Pakistan
9. Turkey
10. Myanmar
11. Afghanistan
12. Yemen
13. Thailand
14. Turkmenistan
15. Uzbekistan
16. Iraq
17. Japan
18. Vietnam
19. Malaysia
20. Oman
21. Philippines
22. Laos
23. Kyrgyzstan

24. Syria
25. Cambodia
26. Bangladesh
27. Nepal
28. Tajikistan
29. North Korea
30. South Korea
31. Jordan
32. Azerbaijan
33. United States Emirates
34. Georgia
35. Sri Lanka
36. Egypt
37. Bhutan
38. Taiwan
39. Armenia
40. Kuwait
41. Timor-Leste
42. Qatar
43. Lebanon
44. Israel
45. Palestine
46. Brunei
47. Bahrain
48. Singapore
49. Maldives
50. Hong Kong
51. Macao

Table S3. Data extraction summary.

Author/Aims	Study details	Participant details	Outcomes measured	Findings	Author Recommendations
**Akerman et al (2017)** To explore healthcare seeking behaviour in relation to sexual and reproductive health and their views on HIV prevention.	**Design/methods:** In-depth interviews **Setting:** Stockholm, Sweden. **Recruitment:** Purposive sampling; language schools and Thai workplaces **Ethics:** Yes	**Inclusion criteria:** Women over 18 and less than 50 years; born in Thailand; lived in Sweden less than 5 years **Sample:** 19 **Response rate:** NA	Attitudes and perceptions of health; experiences with health system; thoughts on HIV prevention.	• Poor access to healthcare in Sweden and wanting to access health care in Thailand • Partners play a key role in access to healthcare • No perceived risk of HIV, but positive attitudes towards prevention.	• Provide cultural competency training for healthcare workers • Integrate HIV testing and contraceptive counselling into cervical screening • Provide free health examinations
**Akerman et al (2016)** To investigate the association between knowledge and utilization of sexual and reproductive healthcare services.	**Design/methods:** Cross-sectional survey; self-report **Setting:** Sweden. **Recruitment:** Postal questionnaire **Ethics:** Yes	**Inclusion criteria:** Women over 18 and less than 64 years; born in Thailand; immigrated to Sweden between 2006-2011. **Sample:** 804 **Response rate:** 62.3%	Contraceptive and STI prevention knowledge; knowledge on health care system; utilization of health care.	• 25% of women had tested for HIV • Low SRH knowledge associated with low access to healthcare • Social capital significantly associated with knowledge about SHR healthcare services	• Provide free health examinations
**Burchard et al (2011)** To understand sexual health knowledge and practices	**Design/methods:** Focus groups **Setting:** Australia **Recruitment:** Purposive sampling at student centre **Ethics:** Yes	**Inclusion criteria:** Born in China; international student; unmarried; or Malaysia **Sample:** 21 **Response rate:** NA	Sexual health and contraception knowledge; recommendations on sexual health education.	• Poor sexual health knowledge • Complex attitudes towards premarital sex • Difficultly accessing sexual health information • Poor understanding of the role of GPs	• Develop of culturally appropriate education programs, which include the role of GPs
**Burke et al (2015)** To explore understandings of HPV vaccine and decision-making around vaccinating their daughter	**Design/methods:** In-depth interviews **Setting:** USA **Recruitment:** Social networks **Ethics:** Yes	**Inclusion criteria:** Cambodian; mother to at-least one girl aged 9-17 years **Sample:** 25 **Response rate:** NA	Understanding of health and prevention; experiences with health care; experiences with HPV vaccination.	• Experiences of being vulnerable (mistreated by relatives, limited food and education)	• No recommendations

				• HPV vaccination viewed as 'protecting' daughter<br>• Confusion on what and how HPV vaccination can 'protect' daughter	
**Dhar et al (2017)**<br>To explore sexual and reproductive health attitudes and beliefs.	**Design/methods:** In-depth, semi-structured interviews<br>**Setting:** USA<br>**Recruitment:** Community events<br>**Ethics:** Yes	**Inclusion criteria:** Aged 16-20 years; Bhutanese refugees; never married; never pregnant<br>**Sample:** 14<br>**Response rate:** NA	SRH knowledge and attitudes; knowledge of SRH services	• Cultural norms influence attitudes towards SRH<br>• Pre-marital sex stigmatised<br>• Misconceptions about rights to access SRH<br>• Gender roles influence SRH	• Provide education on consent and confidentiality as routine part of health care
**Gagnon et al (2010)**<br>To explore how gender-disparities in decision-making affect knowledge, attitudes and practices of STI prevention.	**Design/methods:** Cross-sectional survey<br>**Setting:** Canada<br>**Recruitment:** Through hospitals (post-partum) and community recruitment.<br>**Ethics:** Yes	**Inclusion criteria:** Born in India, Sri Lanka, Pakistan or Bangladesh; able to speak English, French, Hindi, Urdu or Tamil<br>**Sample:** 81<br>**Response rate:** Not reported	HIV and STI knowledge, attitudes and practices; sexual relationship power.	• 50% had never heard of STIs<br>• Higher decision-making power associated with STI knowledge; not keeping HIV a secret; and self- efficacy to negotiate safe sex with partner	• Test public health interventions to increase knowledge, and women's sense of power
**Hawa et all (2019)**<br>To assess HIV risk context and strategies for HIV education and prevention	**Design/methods:** In-depth interviews<br>**Setting:** Canada<br>**Recruitment:** Non-probability, purposive sampling; though community-organisation and snowball sampling.<br>**Ethics:** Yes	**Inclusion criteria:** Adult; self-identifying as South Asian; living with HIV<br>**Sample:** 12<br>**Response rate:** NA	Immigration experience; HIV exposure; perceived barriers to HIV prevention knowledge.	• Stigma a barrier to HIV disclosure<br>• Risk of HIV due to partner's infidelity<br>• Lack of knowledge of HIV<br>• Resistance to condom use	• Offer SRH education in different languages via clinicians; make referrals to support groups; offer HIV testing; offer condoms and lubricant.
**Inoue et al (2016)**<br>To explore understanding and experiences of contraception	**Design/methods:** In-depth; open ended interviews<br>**Setting:** Australia<br>**Recruitment:** Snowball sampling<br>**Ethics:** Yes	**Inclusion criteria:** Aged 16-49; born in Japan; ever used contraception.<br>**Sample:** 7<br>**Response rate:** NA	Knowledge and experiences of contraceptives.	• Preference for condom and withdrawal method as contraception<br>• Difficulties discussing contraception with GP	• Provide information about SRH and healthcare services in community-based media

				• STIs not discussed with partners or GP	• Develop an online website in language<br>• Utilise interpreters in GP consultations<br>• Provide education programs for Japanese students
**Khoei et al (2008)**<br>To explore the meanings of sexuality	**Design/methods:** Interviews and focus groups<br>**Setting:** Australia<br>**Recruitment:** Purposive, volunteer and snowball sampling<br>**Ethics:** Yes	**Inclusion criteria:** Born in Iran<br>**Sample:** 51<br>**Response rate:** NA	Meanings of sexuality, sexual obedience and religion	• Religion had significant effect on women's understanding of sexuality<br>• Feminine sexuality important for marital life<br>• Sexual obedience important religious duty	• No recommendations
**Lindblad et al (2008)**<br>To explore experiences of degrading attitudes related to origin and physical appearance	**Design/methods:** Interviews<br>**Setting:** Sweden<br>**Recruitment:** Adoptee websites<br>**Ethics:** Not reported	**Inclusion criteria:** Aged 18-35 years; adopted from South Korea or Thailand.<br>**Sample:** 17<br>**Response rate:** NA	Experiences of degrading attitudes; perpetrators; strategies for coping with degrading attitudes.	• Experiences of degrading sexual attitudes included exotic associations, men's expectations that women were sexually obedient, and sexual exploitation of women.	• Provide education and support for adoptive parents and children, including through a support group
**Micollier (2017)**<br>To explore the intersection of gender norms and roles, femininity and HIV risk in the context of transcultural spaces	**Design/methods:** Semi-structured, in-depth interviews<br>**Setting:** China and Canada<br>**Recruitment:** Chinese community networks, media and word of mouth<br>**Ethics:** Not reported	**Inclusion criteria:** Lived in Canada for at least one year; born in China.<br>**Sample:** 31 (narratives of 3 women)<br>**Response rate:** NA	Experiences of gender norms; attitudes towards femininity; HIV risk.	• Migrants develop gender-specific transnational lives<br>• Gender power relations favoured men<br>• Men engaged in extramarital sexual relationships, increasing HIV vulnerability for women	• No recommendations

**Nguyen et al (2012)**<br>To explore knowledge of HPV; potential acceptance of the vaccine and factors associated with acceptance, including message framing.	**Design/methods:** Cross-sectional; self-complete paper survey<br>**Setting:** USA<br>**Recruitment:** Community event on breast cancer<br>**Ethics:** Yes	**Inclusion criteria:** Aged over 18; Chinese; ability to read in Chinese<br>**Sample:** 162<br>**Response rate:** 59%	Knowledge of HPV and HPV vaccination; attitudes towards HPV vaccination	• 19% of women had heard of HPV vaccine<br>• Low acceptance of HPV vaccine for daughters (31%)	• Provide educational materials to be published in language<br>• GPs to play a role in encourage HPV vaccine uptake
**Pell et al (2006)**<br>To compare working conditions, migration status and health awareness between 1993-1995 and 2003.	**Design/methods:** Interview-assisted cross-sectional survey<br>**Setting:** Australia<br>**Recruitment:** At clinics and outreach, through Thai and Chinese speaking health education officers<br>**Ethics:** Yes	**Inclusion criteria:** Asian sex workers<br>**Sample:** 91 (1993) and 165 (2003)<br>**Response rate:** Not reported	Sexual behaviour; condom use; knowledge of HIV and STIs.	• 48% sex workers prior to arriving in Australia<br>• Consistent condom use increased for vaginal (52% vs. 85%) and anal sex (20% vs. 78%)	• No recommendations
**Raj et al (2005)**<br>To assess the relationship between IPV and sexual and reproductive health outcomes.	**Design/methods:** Cross-sectional survey and in-depth interviews<br>**Setting:** USA<br>**Recruitment:** Community outreach (fliers, snowball sampling and referrals) for survey; through an organisation for interviews<br>**Ethics:** Yes	**Inclusion criteria:** South Asian, in a heterosexual relationship (survey). History of IPV (interviews).<br>**Sample:** 210 (survey) and 23 (interviews)<br>**Response rate:** NA	Experiences of IPV; SHR outcomes.	• 21% reported physical or sexual abuse by current male partner; 15% in the last 12 months<br>• Significant association between IPV and negative SRH outcomes, including unwanted pregnancies	• Prioritise SHR services among women experiencing IPV<br>• Increase pap smear screening among women
**Richters (2008)**<br>To explore concepts of sexuality and health.	**Design/methods:** Interviews and focus groups<br>**Setting:** Australia<br>**Recruitment:** Volunteer and snowball sampling<br>**Ethics:** Yes	**Inclusion criteria:** Iranian<br>**Sample:** 51<br>**Response rate:** NA	Conceptualization of sexual health	• Sexual health conceptualized mostly as reproductive health<br>• Limited number of non-offensive words for sexual health in Farsi<br>• Low awareness of sexual health services	• No recommendations

**Verran (2015)**<br>To examine how experiences and decisions about family-planning are shaped by cultural background.	**Design/methods:** Semi-structured interviews<br>**Setting:** UK<br>**Recruitment:** Through a Mandarin interpreter at a specialist baby clinic<br>**Ethics:** Yes	**Inclusion criteria:** Originally from China; Mandarin; experience of childbearing.<br>**Sample:** 10<br>**Response rate:** NA	Knowledge and attitudes towards family planning.	• Negative attitudes towards hormonal contraceptives<br>• Cultural preference for male child, positive attitudes towards being able to have multiple children	• No recommendations
**Wray (2014)**<br>To investigate accounts of sexual health including how do women construct sex before marriage.	**Design/methods:** In-depth interviews<br>**Setting:** Australia<br>**Recruitment:** University classes and snowball sampling<br>**Ethics:** Yes	**Inclusion criteria:** Not reported<br>**Sample:** 10<br>**Response rate:** NA	Perceptions towards and experiences of sex before marriage, spouse selection and contraception use.	• Emphasis on female 'purity' (i.e. virginity) and sexual naivety<br>• Segregation from males, females need to be protected<br>• Justification of violence against 'loose' women	• Provide sexual health education as part of early resettlement services

GP: General practitioner, HPV: Human papillomavirus, IPV: Intimate partner violence, NA: Not applicable, SRH: Sexual and reproductive health, UK: United Kingdom, USA: United States of America.
